# Transcriptome Analysis Based on RNA-Seq in Understanding Pathogenic Mechanisms of Diseases and the Immune System of Fish: A Comprehensive Review

**DOI:** 10.3390/ijms19010245

**Published:** 2018-01-15

**Authors:** Arun Sudhagar, Gokhlesh Kumar, Mansour El-Matbouli

**Affiliations:** 1 Clinical Division of Fish Medicine, University of Veterinary Medicine, Vienna 1210, Austria; Arun.Sudhagar@vetmeduni.ac.at (A.S.); Gokhlesh.Kumar@vetmeduni.ac.at (G.K.); 2 Central Institute of Fisheries Education, Rohtak Centre, Haryana 124411, India

**Keywords:** fish disease, immune system, next-generation sequencing, transcriptome

## Abstract

In recent years, with the advent of next-generation sequencing along with the development of various bioinformatics tools, RNA sequencing (RNA-Seq)-based transcriptome analysis has become much more affordable in the field of biological research. This technique has even opened up avenues to explore the transcriptome of non-model organisms for which a reference genome is not available. This has made fish health researchers march towards this technology to understand pathogenic processes and immune reactions in fish during the event of infection. Recent studies using this technology have altered and updated the previous understanding of many diseases in fish. RNA-Seq has been employed in the understanding of fish pathogens like bacteria, virus, parasites, and oomycetes. Also, it has been helpful in unraveling the immune mechanisms in fish. Additionally, RNA-Seq technology has made its way for future works, such as genetic linkage mapping, quantitative trait analysis, disease-resistant strain or broodstock selection, and the development of effective vaccines and therapies. Until now, there are no reviews that comprehensively summarize the studies which made use of RNA-Seq to explore the mechanisms of infection of pathogens and the defense strategies of fish hosts. This review aims to summarize the contemporary understanding and findings with regard to infectious pathogens and the immune system of fish that have been achieved through RNA-Seq technology.

## 1. Introduction

Fish endures as an important source of food and nutrition for hundreds of millions of people worldwide, and aquaculture plays a crucial role in meeting the global food demand [1]. Because of the intensification of aquaculture practices, pathogens pose a major threat to the economic sustainability of the industry. Furthermore, disease outbreaks among feral fish populations are also a major concern especially as natural water bodies deteriorate as a result of anthropogenic activities and climatic changes that affect fish well-being. In order to minimize the impact of diseases in fishes, a knowledge and understanding of the immune defense mechanisms of fishes against pathogens is essential [2]. Moreover, there has been an increasing interest in the investigation of the molecular mechanisms of the immune defense of fish species against pathogens.

RNA sequencing (RNA-Seq) is an evolving modern technology that makes utilization of next-generation sequencing (NGS) or deep sequencing to deal with transcriptome profiling. Transcriptome analysis is a formidable tool that is used for a better understanding of the underlying pathways controlling cell fate, development, and disease progression in a host [3]. Also, it is a rapid and effective approach for genome survey, and massive functional gene and molecular markers identification [4]. In recent years, biological investigations utilizing RNA-Seq have revolutionized and reoriented the outlook, and accelerated the knowledge of the eukaryotic transcriptome.

In the past two decades, the field of transcriptomics has evolved tremendously from the initial studies performed using the hybridization-based microarray technology. Microarray technology has its own limitations, such as the requirement of a prior knowledge of gene sequences from the organism of interest, the occurrence of artifacts caused by cross-hybridization, and the inability to accurately quantify low as well as high expression levels of genes [5,6]. After microarray technology, tag-based sequencing methods, such as series analysis of gene expression (SAGE) and cap analysis gene expression (CAGE), were used for transcriptome analysis, and in these tag-based methods the quantification of genes was more precise than with microarrays. However, tag-based sequencing methods have their own disadvantages, such as the laborious methodology, the requirement of large quantities of input RNA, the high cost of sequencing, the limitations in the quantification of spliced isoforms, and they could not discover novel genes [7].

RNA-Seq technology, with its relatively superior advantages, has slowly replaced microarray and tag-based transcriptome analyses. The greatest advantage of RNA-Seq is that it is both qualitative and quantitative and, hence, it could measure the expression levels of even low-abundance transcripts. Furthermore, it allows the quantification of the expression levels of isoforms [8]. Unlike microarray, this technology has offered the opportunity to generate transcriptomes in almost any non-model organisms of interest, even if the reference gene sequences are unavailable. It is relatively cheaper and enables a better coverage as well as resolution than the methods previously used for transcriptome analysis. As the cost of sequencing is reducing day by day, RNA-Seq facilitates the investigation of more technical and biological replicates of a single study [9]. Apart from polyA mRNA, RNA-Seq could be used to investigate total RNA, pre-mRNA, and various non-coding RNAs [7]. These advantages have made RNA-Seq a better choice for researchers who deal with transcriptomes.

RNA-Seq has already been employed in several studies to understand diseases caused by pathogens in fish and the defense mechanisms of fish against these pathogens. The transcriptome analysis during host–pathogen interaction aids in understanding the changes in the host cells during the course of infection [10]. Particularly, it can reflect the host immune strategies that are activated against the pathogen and also illustrates how the pathogen overcomes host-mediated immune responses directed against it. This information may help to develop effective targeted therapeutics against pathogens. This review specifically outlines the recent research works that use RNA-Seq technology to unravel the mechanisms of infectious diseases and the immune reactions in fish.

## 2. RNA-Seq Workflow

**Figure 1:**
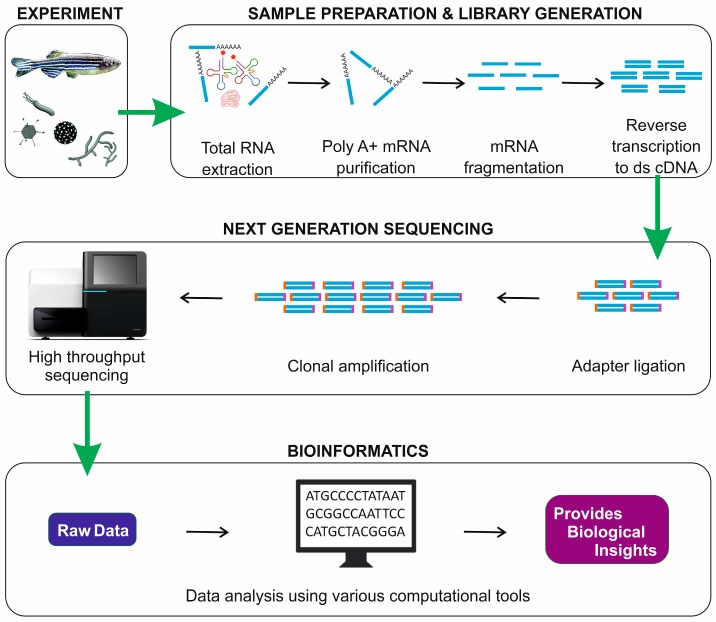
Illustration demonstrating the stepwise workflow of a conventional RNA-Seq-based transcriptome analysis. Four major steps are involved: (i) Experimental design; (ii) Sample preparation and library generation; (iii) Next-generation sequencing of the library; (iv) Bioinformatic analysis.

Major steps involved in a transcriptome analysis using RNA-Seq are: experimental design, library construction, sequencing of the library on a NGS platform, and analysis using bioinformatic tools for interpretation [11]. In this section, we briefly explain the steps involved in RNA-Seq (Figure 1).

The experimental design is an important criterion for RNA-Seq-based transcriptome research. A poor experimental design may result in worthless data along with wastage of ample amounts of money and time. Hence, it is important to design the experiment with the most care and so to fulfill the research objectives. Sequencing depth, desired coverage across transcriptome, and the number of biological and technical replicates must be considered while designing the experiment. Another important thing to be considered is the type of tissue or cell population, which depends on the goals of the experiment. Conventional RNA-Seq experiments use tissue samples containing hundreds of unique cells which are heterogeneous in nature, and the results observed are confounding [12,13]. To overcome this problem, a transcriptome analysis of single cells could be done, and, to this end, many single cell isolation protocols have been established in recent times [13]. However, the major challenge observed in single-cell RNA-Seq (ScRNA-Seq) is the extremely low RNA concentration available for the analysis. In addition to this, it is important to note that the transcriptome analysis of heterogeneous tissue samples helps in discovering novel pathways and the cellular origin of a disease [14,15]. Hence, the choice of the biological sample for RNA-Seq-based transcriptome analysis for either single cell or heterogeneous tissues is performed based on the objectives of the researcher.

After the experimental design, the next step is the RNA preparation and library construction, which starts with total RNA isolation from the tissue or cell of interest. It is important to check the quality of RNA before proceeding to the further downstream step, and this can be done by using the Agilent Bioanalyzer, which provides representative values as RNA integrity numbers (RIN) lying between 1 and 10. A high RIN value represents RNA of high quality with very little degradation. Typically, RIN values lower than 6 may lead to misleading results. Total RNA is comprised of rRNA, pre-mRNA, mRNA, and various ncRNA. After the quality check, it is important to separate the particular RNA species of interest from total RNA, depending on the research objectives. The total RNA samples could be either enriched or depleted in order to obtain particular species of RNA. Poly-A selection and enrichment are done for sequencing mRNA; ribo-depletion is performed for the sequencing of mRNA, pre-mRNA, and ncRNA; size-selection strategy is followed for miRNA sequencing [7]. The RNA molecules are then either fragmented and reverse transcribed to double-stranded cDNA, or first reverse transcribed to double-stranded cDNA and then fragmented [8,16]. The fragmentation is done in order to increase the coverage during the sequencing process, and all RNA species do not require the fragmentation process. Both ends of the fragmented cDNA are then ligated with sequence adapters and are now ready for sequencing [17].

There is a rapid development and improvement of NGS technologies day by day, which fine-tune the sequencing chemistry and make the technology more affordable. Illumina, Ion Torrent, and PacBio are the most commonly available sequencing platforms in the market [18,19]. Among them, in recent times, Illumina-based platforms are most preferred among the researchers. Each platform adopts different adapter sequences and different sequencing chemistries [17]. The choice of the sequencing platform which provides either short-read sequences or long-read sequences has a significant role in the transcriptome assembly. Long sequence reads (>1000 bp) are preferred for de novo assembly which is easy to assemble and advantageous in identifying splicing isoforms. On the other hand, short sequence reads (50–100 bp) have a relatively higher coverage and a lower error than long-read sequences [7]. The sequence reads are generated as FASTQ-format files from the NGS platform and are further used for bioinformatics analyses.

**Figure 2:**
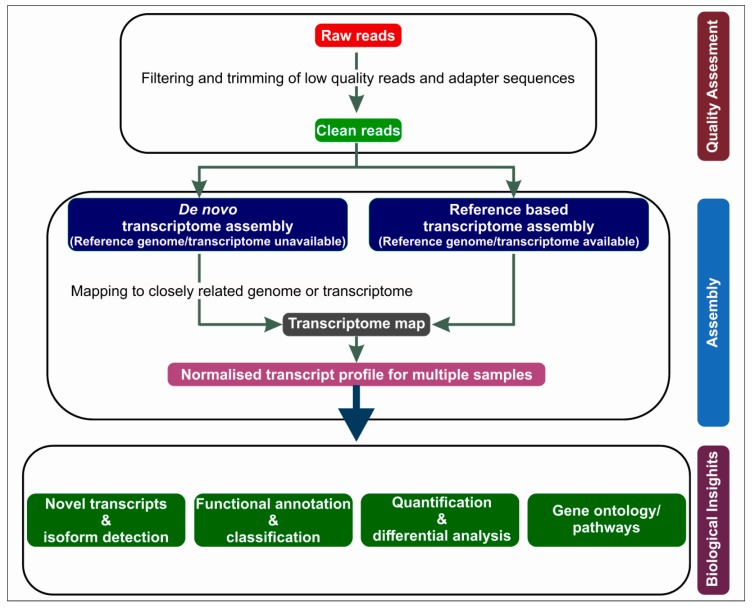
Schematic representation of the processing of data obtained from next-generation sequencing (NGS), by means of bioinformatics. Briefly, the raw reads obtained from NGS are subjected to quality assessment, and high-quality clean reads are further processed for transcriptome assembly. The assembled normalized transcriptome could be further explored for biological insights.

The bioinformatics analysis of RNA-Seq (Figure 2) starts with the refinement of the sequence reads, which involves the removal of the adapter sequences, the trimming and discarding of reads based on quality, and the filtration of the sequence reads based on K-mer coverage. This is then followed by the assembly of the filtered sequence reads (de novo assembly) or by the alignment of the sequence reads to a reference genome (re-sequencing). The filtered reads are subjected to de novo assembly and annotation in case of nonavailability of a reference genome. If a reference genome is available, the filtered read sequences could be assembled over it. Various choices of alignment tools and assemblies that are relatively well standardized are available. The assembled or aligned sequences could be utilized for the quantification of transcripts and their isoforms [20,21]. TOPHAT, RUM, STAR, GSNAP, GEM, MAPslice etc. are some of the bioinformatics tools used for read alignments; Cuflinks, iRreckon, FluxCapacitor etc. could be employed for transcriptome assembly and quantification; differential expression analyses could be performed by employing tools such as DESeq, DEGseq, Cuffdiff, Bayseq, EdgeR etc. These bioinformatics tools continue to develop day by day in order to meet new challenges in RNA-Seq. The desired choice of bioinformatics tools is always based on a researcher’s preferences and research objectives.

## 3. Fish Immune Response to Bacterial Pathogens

Studies involving RNA-Seq technology to understand fish diseases and pathogen evasion strategies against the immune system have been performed mostly during bacterial infection (Table 1). Zebrafish (*Danio rerio*) is the most commonly used fish species and an excellent research model. Zebrafish embryos challenged with *Salmonella typhimurium* were utilized to understand the role of Traf6 function in the innate immune response [22] and transcriptome changes during the course of infection [23]. Similarly, immune-responsive actions of the muscle tissue were revealed in zebrafish during *Mycobacterium marinum* (*M. marinum*) infection [24]. *M. marinum* infection in common carps (*Cyprinus carpio*) revealed 39 TIR domains with transcript isoforms and genes [25]. *M. marinum* infections in fish are used as a model to understand tuberculosis disease in humans caused by *Mycobacterium tuberculosis.*

Enteric septicemia of catfish is caused by *Edwardsiella ictaluri* (*E. ictaluri*) and is an economically important disease which affects the catfish aquaculture industry. Investigations on the pathogen entry mechanisms and the mucosal immune response in channel catfish (*Ictalurus punctatus*) showed the importance of the actin cytoskeletal polymerization and remodeling, and of the junctional regulation of *E. ictaluri* entry by intestinal barrier disruption [26]. Furthermore, in order to gain a profound understanding, RNA-Seq data from previous studies were utilized to further investigate nucleotide-binding oligomerization domain like receptors (NLR) [27], retinoic acid-inducible gene I [28], Rab GTPase [29], and Toll-like receptors (TLR) [30] in channel catfish during *E. ictaluri* infection. A bulk segregant RNA-Seq analysis of the F2 generation of interspecific hybrids of channel catfish and blue catfish (*Ictalurus furcatus*), which are respectively, relatively susceptible or extremely resistant to *E. ictaluri* infection, showed a higher expression of immune-related genes in the resistant fish than in the susceptible fish [31]. In addition, biomarkers contributing to the genetic resistance of the yellow catfish (*Pelteobagrus fulvidraco*) to *E. ictaluri* were established using RNA-Seq [32]. A total of 24 hub genes involved in protein–protein interactions were identified from the gill transcriptome of the Japanese flounder (*Paralichthys olivaceus*) infected with *Edwardsiella tarda* [33].

RNA-Seq was used to understand the immune evasive mechanisms of *Flavobacterium columnare* (*F. columnare*) which targets mucosal organs such as the gill and the skin, used as routes of entry. The strain of channel catfish susceptible to *F. columnare* infection showed a basal polarization of gill mucosal compartments possessing a putative mucosecretory and tolerogenic phenotype, leading to the evasion of the pathogen [34]. Also, a dramatic upregulation of a rhamnose-binding lectin (RBL) in the gill of the channel catfish was observed during *F. columnare* infection [35]. It is interesting to note that RBL has putative roles in bacterial attachment and aggregation. This finding was used to test the protective nature of sugars having putative RBL ligands, such as l-rhamnose and d-galactose, against *F. columnare* infection. The exposure of the channel catfish to l-rhamnose and d-galactose showed a significant protection against the bacteria [36]. In a recent study, channel catfish, immersion-vaccinated and unvaccinated against *F. columnare*, were challenged with the same bacteria, and a comparative transcriptome analysis was done. The results suggested that in the vaccinated fish a strong expression of genes related to collagen deposition and tissue remodeling occured, compared to the unvaccinated group [37]. This clearly indicates that, apart from adaptive immune actions, mucosal vaccines may contribute to innate immune responses and tissue repair.

*Aeromonas hydrophila* is an opportunistic bacterial pathogen of fish found in the freshwater and brackish water environment. Different studies utilized RNA-Seq to investigate *A. hydrophila* infection in fishes of aquaculture importance, such as the large yellow croaker (*Larimichthys crocea*) [39], the blunt snout bream (*Megalobrama amblycephala*) [46], the common carp [52], the grass carp (*Ctenopharyngodon idella*) [51,57], the golden mahseer (*Tor putitora*) [54], and the darkbarbel catfish (*Pelteobagrus vachellii*) [60]. These studies provided a deeper understanding of the defense mechanisms of fishes against *A. hydrophila*. Similarly, RNA-Seq data from a previous study was used to design a customized microarray in order to decipher the mucosal immune defense in the blue catfish during *A. hydrophila* infection [61]. A transcriptome analysis of the Nile tilapia (*Oreochromis niloticus*) during *Streptococcus agalactiae* infection revealed that toll-like receptor-mediated pathways [59] and miRNAs contribute to immune responses which protect the host against the pathogen [47]. In addition to this, tilapia could rapidly recognize the invading bacteria and activate downstream immune signaling pathways to clear the bacteria, thereby preventing tissue damage and bacteria-triggered cell apoptosis [53]. There was an acute inflammatory response and recruitment of macrophages for pathogen clearance in tilapia [44]. Also, the event of global warming could impair the functions of immune-related genes in the Mozambique tilapia (*Oreochromis mossambicus*) and enhance *S. agalactiae* infection [49].

RNA-Seq analysis of the half-smooth tongue sole (*Cynoglossus semilaevis*) [45] and the Japanese sea bass (*Lateolabrax japonicus*) [50] challenged with *Vibrio anguillarum* identified several immune-related pathways and candidate genes during the infection progress. RNA-Seq analysis of the Japanese sea bass during *Vibrio harveyi* challenge revealed unique transcripts, about 8% of which were highly specific to fish and have never been reported before. Also, the study suggested that fish immune system may be more complex than previously believed, and fish might have acquired numerous fish-specific immune system components during early vertebrate evolution [38]. A transcriptome analysis of the orange-spotted grouper (*Epinephelus coioides*) infected with *Vibrio alginolyticus* suggested that the host defense mechanisms may activate 4 h after bacterial infection by means of the complement pathway and the antimicrobial peptide hepcidin [43]. Similarly, transcriptome studies of the orange-spotted grouper challenged with *V. harveyi* provided a deeper understanding of immune-related pathways acting against the bacteria [58]. In the case of the giant grouper (*Epinephelus lanceolatus*) infected with *V. alginolyticus*, it is believed that a TLR 5-mediated regulation leading to cytokine regulation may be involved in the immune response [48]. The Asian sea bass (*Lates calcarifer*) subjected to different stimuli, such as lipopolysaccharide, *V. harveyi* challenge, high salinity, and fasting, showed a global coordinated reaction in response to different stressors and a dramatic mucosal immune response in the intestine [42].

The transcriptome analysis based on RNA-Seq has been employed in few vaccination studies against bacterial infection. Zebrafish subjected to an *E. tarda* live attenuated vaccine showed the upregulation of genes involved in the major histocompatibility complex (MHC)-I pathway, whereas genes associated with the MHC-II pathway were downregulated during early stages of vaccination [41]. The oral vaccination of the European sea bass (*Dicentrarchus labrax*) against *V. anguillarum* led to increased expression of rare transcripts, such as leukocyte immune-type receptors and cullin or supervillin [40]. *Yersinia ruckeri* is a Gram-negative, rod-shaped bacterium which causes enteric redmouth disease in fishes [62]; proteins of the bacteria are often studied to understand its virulence and pathogenicity [63,64]. RNA-Seq-based transcriptome analysis revealed immune-related pathways in the spleen of Amur sturgeon (*Acipenser schrenckii*) during *Y. ruckeri* infection [55] and the importance of the flagellar master operon *flhD* in the virulence of the bacteria [56].

## 4. Fish Immune Response to Viral Pathogens

Viral diseases are a paramount threat to the fish culture industry causing acute and huge economic losses. During the infection process, once a virus spreads beyond few host cells, a variety of nonspecific and specific host defensive mechanisms will be triggered, which will try to contain the virus and minimize host cell damage [65]. Detailed studies were performed to analyze the transcriptomic changes in fish in order to understand the immune responses during viral infections using RNA-Seq technology, as summarized in Table 2.

The grass carp is an economically important candidate species for aquaculture. In recent times, grass carp farming has been severely hampered by grass carp reovirus (GCRV) infection, a dsRNA virus belonging to the *Aquareovirus* genus of the family Reoviridae [69]. GCRV infection leads to a hemorrhagic disease and is highly fatal during the early growth stages of the fish. Three different studies utilized RNA-Seq to investigate GCRV infection in the grass carp. The immune changes in different tissues indicated that GCRV does not target any specific tissue, but disturbes lipid and carbohydrate metabolism in various organs [69]. However, the immune response of the spleen was more intense than that of the head kidney, which provides evidence that alternative splicing plays a pivotal regulatory role in the immune response [71]. Also, a transcriptome analysis combined with RT-PCR validation identified the complement and coagulation cascade pathways as relevant pathways in the defense against GCRV infection [73].

The viral hemorrhagic septicemia virus (VHSV) is a notifiable aquatic animal disease listed by OIE (World Organisation for Animal Health) infecting over 80 freshwater and marine species worldwide [88]. VHSV belongs to the genus *Novirhabdovirus* of the Rhabdoviridae family [89,90] and represents a serious threat to the salmonid aquaculture industry in Europe. In order to decipher the underlying genetic mechanisms of resistance versus susceptibility to VHSV of clonal lines of the rainbow trout (*Oncorhynchus mykiss*), a whole genome transcriptome analysis using RNA-Seq analysis was performed [68]. VHSV was found to suppress metabolic functions such as ATP synthesis and antioxidant systems, leading to necrosis in the host cells [85]. The infectious salmon anemia Virus (ISAV) is a ssRNA virus of the Orthomyxoviridae family which often causes serious disease outbreaks in the salmonid farming industry. ISAV is comprised of 8 RNA segments which code for 10 proteins [91] and typically causes a systemic infection in the endothelial cells of the blood vessels [92]. RNA-Seq analysis in the Atlantic salmon (*Salmo salar*) during ISAV infection suggested that the molecular analysis of viral segments replication has the potential to identify important immune-related genes and their responses during host–pathogen interactions [70,75].

Piscine orthoreovirus (PRV) is a nonenveloped dsRNA virus belonging to the family Reoviridae and causes heart and skeletal muscle inflammation (HSMI) disease in the Atlantic salmon [93]. This virus could replicate in the sockeye salmon but could not cause HSMI [94]. Similarly, the infectious hematopoietic necrosis virus (IHNV) is a bullet-shaped ssRNA virus, member of the Rhabdoviridae, that is a major threat to the North American, European, and Asian fish culture industry [95]. Kidney transcriptome analysis of the sockeye salmon (*Oncorhynchus nerka*) during PRV infection along with superinfection of IHNV suggested that the host invasion strategies of PRV and IHNV are independent of each other during coinfection. Even a robust host antiviral pathway produced during IHNV superinfection did not affect PRV multiplication [79]. This study clearly stated that, during coinfection, PRV and IHNV do not influence each other when invading the host.

Salmonid alphavirus (SAV) is an enveloped ssRNA virus from the Togaviridae family, and its infection in the Atlantic salmon may result in necrosis in the exocrine pancreas, cardiomyopathy, and skeletal myopathy [96]. A transcriptome analysis showed a strong type-I interferon (IFN) response induced by SAV-3 in the macrophage/dendritic-like cell line derived from the Atlantic salmon, and this could be useful in understanding IFN responses in salmonids [80]. Cyprinid herpesvirus-3 (CyHV-3), or koi herpesvirus (KHV), is a dsDNA virus categorized under the genus *Cyprinivirus*, family Alloherpesviridae, order Herpesvirales [97]. KHV is highly contagious and leads to higher rates of mortality in the common carp and koi carp (*Cyprinus carpio* koi) [98]. A spleen transcriptome analysis of the koi carp during CyHV-3 infection identified various novel genes which contribute significantly to the innate immune response against the virus [82]. The spring viremia of carp virus (SVCV), is also a notifiable aquatic animal disease listed by the OIE, which causes acute haemorrhages in cyprinids, leading to mortality. A transcriptome analysis of SVCV in zebrafish illustrated that the virus may lead to a significant inflammatory and metabolic response upon infection in the host [84].

RNA-Seq analysis of the kidney of the olive flounder (*Paralichthys olivaceus*) revealed that heat-inactivated vaccination against VHSV modulates numerous genes involved in the acute phase response, TLR, IFN, and apoptosis pathways [74]. Poly (I:C) is an immunostimulant often used as a viral mimic. A transcriptome analysis of the spleen of the large yellow croaker injected with poly (I:C) showed a considerable amount of immune relevant genes and pathways that are remarkably similar in mammals, which suggests that the mechanisms underlying the innate and adaptive immunity in fish may be conserved among vertebrates [67]. Similarly, poly (I:C) activated miiuy croaker (*Miichthys miiuy*) revealed retinoic acid inducible gene-I like receptors (RLRs), like MDA5 and LGP2, that are involved in host surveillance against infection of dsRNA viruses and induce a type I IFNs response to inhibit virus replication [72]. In another study, RNA-Seq analysis of rainbow trout erythrocytes stimulated with poly (I:C) revealed the role of nucleated erythrocytes of fish in defense mechanisms against viruses, acting through the production of thermolabile molecules that activate the macrophages [66]. Similarly, the liver transcriptome of the yellow catfish induced with Poly (I:C) showed insights in the immune system [83]. RNA-Seq revealed the role of MDA5- and janus kinase (JAK)-mediated signaling pathways in preventing viral invasion [76]. A comparative transcriptomic analysis of the orange-spotted grouper during two different iridovirus infections provided insights about the mechanisms of the immune response in fish related to different viruses belonging to the same group [77].

Similarly, the orange-spotted grouper infected with the Singapore grouper iridovirus (SGIV) had a significant influence on the mitogen-activated protein kinase, chemokines, TLRs, and RIG-I signaling pathways [86]. Megalocytivirus, another virus from the iridovirus family, is known to cause disease outbreaks during the summer season when the temperature is high. Comparative transcriptomics of turbot (*Scophthalmus maximus*) during megalocytivirus infection in low and high temperatures showed that the temperature has a significant role in the global transcription of turbot, which in turn influences the immune system [87]. The study revealed that the megalocytivirus invasion in the host was significantly low at lower temperatures.

Nodaviruses are RNA viruses which are categorized into three groups, i.e., alphanodavirus which infects insects, betanodavirus which infects fishes, and gammanodavirus infecting prawns [99]. The nervous necrosis virus (NNV) is a betanodavirus and is the causative agent of viral encephalopathy and retinopathy (VER) also known as viral nervous necrosis (VNN). This virus has a host range of almost 120 fish species resulting in 100% mortality during larval stages and in juveniles [100]. NNV has developed a mechanism to avoid the host innate and adaptive immune responses [101,102,103]. Transcriptome studies in grouper cell lines during betanodavirus infection showed the role of the viral capsid protein in the endoplasmic reticulum stress response, which may eventually lead to cell death [78]. Similarly, a transcriptome analysis of the leukocytes of the European sea bass (susceptible species) and of the gilthead seabream (*Sparus aurata*) (resistant species) suggested that NNV adopts a mechanism to escape the cell-mediated cytotoxicity, leading to fatal infection in the European sea bass [81]. Furthermore, previous RNA-Seq data were utilized to design customized microarray to study VNN infection in the Atlantic cod (*Gadus morhua*) [104].

Similarly, previous RNA-Seq data from the grass carp during GCRV infection was used in order to check the performance of two downstream bioinformatics tools, i.e., DESeq and Cufflinks, which were used to calculate the differential expression of genes during RNA-Seq data analysis. This study provided insights into two immune-related genes, ISG15 an ubiquitin-like protein and sacsin-like gene which is likely to regulate mitochondrial dynamics in the grass carp [105]. It is clear that RNA-Seq-based transcriptomics analysis is an inevitable tool not only to unveil host–viral interactions in aquatic organisms, but also to help to characterize the viral mechanisms to escape the host defense mechanisms.

## 5. Fish Immune Responses to Parasites

Parasites are a major concern affecting feral fish population as well as cultured fish. All of the major groups of animal parasites were reported in fish, i.e., protozoa, myxozoa, helminthic worms, and crustacean parasites. Parasites use various immune evasion strategies, like avoidance of the immune system surveillance, indirect life cycle, molecular mimicry, immune suppression, immune modulation, and manipulation of the host endocrine and neural systems to invade their hosts [106]. Studies involving RNA-Seq to understand fish parasites are presented in Table 3.

*Cryptocaryon irritans* (*C. irritans*) is an economically important ectoparasite which causes ich disease or cryptocaryoniasis in fish, and RNA-Seq has been used significantly to explore this disease. Immune mechanisms, such as the toll pathway, chemokine signaling, complement system, and coagulation cascades, showed profound responses during the parasite infection in the large yellow croaker [111]. In addition to this, single sequence repeats (SSRs) and single nucleotide polymorphisms (SNPs) explored during this study would be resourceful for markers development, genetic linkage, and QTL analysis. Alternative pathways of the complement system played a major role in the early stages of *C. irritans* infection. It is interesting to note that even a low, nonlethal infection of *C. irritans* could induce a significant immune response in fish; in contrast, an immunosuppression-like condition was noticed in the large yellow croaker with a fatal infection [113]. There was a local immune response with intensive leucocytes migration and TLR-associated pathogen-associated molecular pattern recognition of parasites in the skin of the orange-spotted grouper affected with *C. irritans* [116]. Two studies directly employed RNA-Seq-based transcriptome analysis to study the parasitic stages of *C. irritans* with the objective of identifying potential vaccine candidates. The analysis of the tomont stage of *C. irritans* under low temperature helped in the identification of several genes required for the parasitic stage to enter a deeper dormancy state and survive low temperatures. These genes might be considered as potential candidate genes to develop diagnostic and control measures for cryptocaryoniasis [114]. Similarly, 9 putative immobilization antigens transcripts and 161 protease transcripts were found in the transcriptome of *C. irritans* which could be considered as major targets for vaccines and drug development, respectively [115]. Similarly, transcriptome data obtained from six developmental stages of the salmon louse *Caligus rogercresseyi* as well as from the adult male and female will serve as a valuable tool for future genomic studies to understand host–parasite interactions in the salmon louse [109].

When the three-spined stickleback (*Gasterosteus aculeatus*) was infected with three different genotypes of the trematode parasite *Diplostomum pseudospathaceum,* the genetic variation in parasitic worms influenced the mechanisms by which the host immune system reacted to an immunological threat [108]. In another study involving the same fish and parasite species, the same research group studied the homologous and heterologous exposures of genetically distinct trematode parasite lineages on gene expression patterns of adaptive immunity. It is interesting to note that host immunization was optimized towards an adaptive immune response that favored effectiveness against parasite diversity over specificity [110].

RNA-Seq analysis of the large yellow croaker infected with the intestinal myxozoan parasite *Enteromyxum scophthalmi* (*E. scophthalmi*) showed an exacerbated local immune response in the pyloric caeca, whereas an inadequate activation of the adaptive immunity was observed in the spleen and kidney as a result of the failure in bridging between the innate and adaptive immune responses [107]. Similarly, another study involving RNA-Seq analysis of turbot infected with *E. scophthalmi* showed the vital role of the IFN-mediated immune response during the early phase of the disease [112]. The myxozoan parasite *Tetracapsuloides bryosalmonae* (*T. bryosalmonae*) is the causative agent of proliferative kidney disease which significantly affects both farmed and wild salmonid fishes in Europe and North America, causing significant economic losses, and endangers the wild fish population [117]. There is no RNA-Seq study of *T. bryosalmonae* and its invertebrate host *Fredericella sultana*, and only a few expressed sequence tags have been deposited in the National Center for Biotechnology Information (NCBI) GeneBank database. Recently, it has been demonstrated that *T. bryosalmonae* can complete the development and produce mature spores only in the brown trout but not in the rainbow trout [118,119,120]. However, till now there are no RNA-Seq-based transcriptome studies to understand the detailed fish host evasion strategies against *T. bryosalmonae*.

## 6. RNA-Seq Analysis of Oomycetes

*Aphanomyces invadans* (*A. invadans*) and *Saprolegnia parasitica* (*S. parasitica*) are endemic to freshwater habitats and belong to the water molds or oomycetes group. These two oomycetes are responsible for significant economic losses in the aquaculture industry [121]. *A. invadans* is known to cause the epizootic ulcerative syndrome across fish species and could possibly invade the host by means of some extracellular proteases [122]. Similarly, *S. parasitica* causes the saprolegniasis disease, a major threat to the salmonid farming industry. Only one study has exploited RNA-Seq-based transcriptome analysis to understand *S. parasitica*. RNA-Seq analysis of four life stages and a cell culture-based time-course study revealed that the kinome of *S. parasitica* has a significant function during infection. Furthermore, several putative proteins expressed in preinfection life stages may help the oomycete to interact with the host [123].

## 7. RNA-Seq Analysis of Healthy Fish

Fish is an evolutionarily primitive animal among vertebrates, and the immune system of fish is highly complex. The immune system works continuously to protect the host from invading pathogens. Transcriptome analysis based on RNA-Seq is now being used to understand the immune system and its evolutionary significance in normal fish without infections (Table 4). The immune organs of fish are slightly different from those of other higher vertebrates, and with the exception of the lymph nodes and bone marrow, most immune organs of mammals are found in fish [124]. Kidney, spleen, thymus, and gut-associated lymphoid tissue (GALT) are the immune organs present in fish [125]. Most RNA-Seq transcriptome analyses use kidney and spleen as the organs of choice to decipher the immune mechanisms during host–pathogen interaction. Apart from the primary immune organs, RNA-Seq revealed the expression of immune genes in other tissues, such as the gill, swim bladder, skin, brain, muscle, testis, ovary, heart, liver, and pancreas. An analysis of the spleen of the rainbow trout using RNA-Seq technology suggested that innate and adaptive immune systems of fish are highly conserved among vertebrates [126]. Fish species like the Japanese flounder [2], the naked carp (*Gymnocypris przewalskii*) [127], the schizothoracine fish (*Gymnocypris przewalskii ganzihonensis*) [128], the topmouth culter (*Culter alburnus*) [129], and the grass carp [130] were studied to understand immune-related genes and pathways using RNA-Seq technology. RNA-Seq revealed the evolutionary relatedness between the fish swim bladder and the mammalian lung, along with the involvement of five immune-related pathways in the swim bladder of fugu (*Takifugu rubripes*) [131]. Similarly, in tilapia, during the process of evolution, two immune-related genes, i.e., Notch2, known for its role in cell differentiation and in the regulation of dendritic cells, and nfatc3b, involved in thymocyte differentiation, were positively selected by the environment [132]. Also, RNA-Seq studies explored an active subpopulation of T lymphocytes in the gill tissue of the European sea bass [133] and a restricted subset of T helper cells in the elephant shark (*Callorhinchus milii*) [134]. Transcriptome data obtained from the ayu fish (*Plecoglossus altivelis*) were employed to design a customized microarray, which was used to test the effects of recombinant LECT2 on the macrophages of ayu fish [135]. Recently, scRNA-Seq of kidney-derived blood cells of zebrafish revealed that haematopoiesis is highly conserved between fish and higher vertebrates [136]. Also, this scRNA-Seq technology was used to study immune cell types from the spleen of zebrafish, which showed the evolution of immune cells in lower vertebrates [137].

Mucosal organs, such as the gut, skin, and gill, are constantly exposed to the external environment and could be considered as the main route for pathogen entry in fish [140]. Hence, the imperilment of the mucosal immune defense may lead to pathogenic invasion and disease in fish. RNA-Seq-based transcriptome profiling was used as an effective tool to understand the mucosal immune system in the skin of the mud loach (*Misgurnus anguillicaudatus*), which revealed a complex immune mechanism involving chemokine signaling, leukocyte transendothelial migration, and T cell receptor signaling [138]. Similarly, in the channel catfish, an altered immune status in the surface mucosa was found after short-term starving, leading to pathogen invasion [141]. Nutrition has an important role in the overall development and well-being of an organism. A transcriptome analysis in various larval stages of zebrafish revealed that sufficient nutrition supplementation is highly essential for the activation of antigen processing and presentation pathways during the early stages of larval development [139].

Abiotic factors, such as temperature, crowding, salinity change, sunlight, and oxygen levels, are found to exhibit a significant influence on the immune system of fish. RNA-Seq-based transcriptome profiling unveiled alterations in immune genes of the crimson-spotted rainbowfish (*Melanotaenia duboulayi*) under high-temperature stress [142], of the olive flounder under low-temperature stress [143], of the rainbow trout under handling and confinement stress [144], and of the large yellow croaker under high-stocking stress [145]. Under hypoxic conditions, a transcriptome analysis of the brain in the large yellow croaker revealed a network of neuroendocrine–immune system that prevents inflammation in the cerebrum of the brain and regulates energy balance during low oxygen levels in water [146]. Similarly, under high-salinity conditions, the immune responses were found to be suppressed in the Japanese eel (*Anguilla japonica*) when compared to lower salinity [147]. Lipopolysaccharide (LPS) is considered to be a potential immunostimulant and it is the main component of the Gram-negative bacterial cell wall. The yellow catfish stimulated with LPS showed a significant upregulation in the nonspecific immune responses, such as CXCL2-like chemokine, goose-type lysozyme, and cathepsin K [148]. Plant-based immunostimulants are considered to be an effective prophylactic measure to prevent diseases by enhancing the nonspecific immune system of fish [149]. RNA-Seq was used to evaluate the immunoregulatory properties of three Chinese herbs, namely, *Spatholobus suberectus*, *Phellodendron amurense*, and *Eclipta prostrate*, after dietary supplementation to the tiger grouper. The experiment interestingly revealed that *S. suberectus* was immunostimulatory, whereas *P. amurense* and *E. prostrate* were immunosuppressive [150]. This was the first study to evaluate the effectiveness of herbal immunostimulants in fish using RNA-Seq-based transcriptome analysis. It is clear that RNA-Seq-based transcriptome analysis not only gives insights into the immune system of a healthy fish, but also helps to evaluate the performances of various factors and stimuli which influence the immune system. In addition, it may help to understand the evolution of the immune system among vertebrates.

## 8. Conclusions and Future Directions

In this review, we have summarized the applications of RNA-Seq technology for a deeper understanding of pathogenic diseases and defense mechanisms in fish. Being qualitative as well as quantitative, this technology has led to the identification of many molecular actors and their patterns of expression in a limited time-frame, laying the foundation for further studies in the mechanisms of host–pathogen interaction. This technology has also aided in testing the efficacy of vaccines [41,44,74], treatment options [107,114], immunostimulants [150], and immunoprotective diets [36] against pathogens. Transcriptome data derived during host–pathogen interactions will be useful to understand virulence genes of the pathogens that are induced in vivo. These novel genes will support the development of effective therapeutics and vaccines [112]. Moreover, SNPs and SSRs discovered in RNA-Seq transcriptome datasets may aid with genetic linkage mapping, quantitative trait analysis, and pathogen-resistant strain or broodstock selection [2,31,45,109,111]. RNA-Seq studies have not only provided a large number of candidate genes, but they have also helped to enhance the contemporary knowledge of immunogenetics and the evolution of immune-related genes and their families [127].

Apart from this, RNA-Seq-based transcriptome analysis of fish has its own limitations compared to its mammalian counterpart. During evolution, genome duplication has determined a large variation in the genes of fish compared to other animal groups [132], and the presence of multiple isoforms of genes is the major challenge encountered during RNA-Seq data analysis. Bioinformatics algorithms are available to analyze splicing events of transcripts, but because of the presence of multiple copies of various genes, this analysis is very challenging in fish. These gene variants may show different ontology as well as expression and are often challenging to interpret [9].

The use of RNA-Seq to analyze transcriptome changes started at the end of the last decade and, day by day, its application in the field of fish health is in the log phase. This technique is highly dynamic and evolves based on new challenges and research objectives. In the past few years, ScRNA-Seq protocols have gained momentum, rendering transcriptome information from a single cell and thus avoiding the limitations caused by heterogeneous cell populations [151]. However, the requirement of a minimal amount of genetic material and the possible sample loss, and contamination make ScRNA-Seq more challenging than conventional RNA-Seq. Traditionally, protein-coding poly-A mRNA is used for transcriptome analysis based on RNA-Seq, and, currently, researchers are showing more interest in investigating other RNA species, including total RNA, pre-mRNA, and different types of ncRNAs, such as miRNA, siRNA, piRNA, snoRNAs, snRNA, lncRNA, and circRNA. Most small ncRNA are known to be involved in post-transcriptional regulation of gene expression [152] and may be linked to immune functions as well as to the suppression of diseases of the host. More research has to be focused on these unconventional RNA species which may contribute to the current understanding of the immune function in fish. Moreover, targeted investigations have to be focused to enhance the current knowledge of fish pathogens and their defense strategies during disease progression. It is evident that RNA-Seq technology is a time-saving promising tool in the field of fish health.

## Figures and Tables

**Table 1:** RNA-Seq analysis of fish in response to bacterial pathogens. This table shows a description of the transcriptomes to understand bacterial diseases in various fish species.

Sl No	Fish	Bacterial Species	Sample	Sequencing Platform	Sequencing Depth/Library	Accession Number	Reference
1	Japanese sea bass	*Vibrio harveyi*	Head kidney & spleen	Illumina Genome Analyzer II	Control: 33.03 M	GSE21721	[38]
					Challenged: 34.59 M		
2	Zebrafish	*Salmonella typhimurium* containing DsRed vector	Whole embryo	Illumina Genome Analyzer II	15 M ^a^	GSE21024	[22]
3	large yellow croaker	*Aeromonas hydrophila*	Spleen	Solexa/Illumina Genome Analyzer	13.6 M	SRA010789	[39]
4	Zebrafish	*Salmonella typhimurium*	Whole embryo	Illumina Genome Analyzer II	15 M	GSE21024	[23]
5	Common carp	*Mycobacterium marinum*	Whole embryo & adult animals	Illumina Genome Analyzer II	NA	NA	[25]
6	European sea bass	Oral vaccination against *Vibrio anguillarum*	Head kidney & hind gut	Genome Sequencer FLX Titanium	0.269 M	SRA050000	[40]
7	Zebrafish	*Edwardsiella tarda* live attenuated vaccine	Liver	Illumina HiSeq 2000	Control: 18.13 M ^a^ Vaccinated: 14.27 M ^a^	SRA048658	[41]
8	Channel catfish	*Edwardsiella ictaluri*	Intestine	Illumina HiSeq 2000	197.6 M	SRP009069	[26]
9	Channel catfish	*Flavobacterium columnare*	Gill	Illumina HiSeq 2000	203.2 M	NA	[35]
10	Channel catfish	*Flavobacterium columnare*	Gill	Illumina HiSeq 2000	350 M	SRP017689	[34]
11	Hybrid of channel catfish & blue catfish	*Edwardsiella ictaluri*	Liver	Illumina HiSeq 2500	400 M	SRP028159	[31]
12	Asian seabass	*Vibrio harveyi*	Intestine	Genome Sequencer FLX Titanium	1.0 M	DRR002185–DRR002190	[42]
13	Orange spotted grouper	*Vibrio alginolyticus*	Whole animal	Illumina HiSeq 2000	114.8 M *	GSE63148	[43]
14	Tilapia	*Streptococcus iniae*	Spleen	Illumina HiSeq 2000	223 M	NA	[44]
15	Half-smooth tongue sole	*Vibrio anguillarum*	Liver, head kidney, spleen & intestine	Illumina HiSeq 2000	80.8 M	NA	[45]
16	Blunt snout bream	*Aeromonas hydrophila*	Blood, liver, gill, intestine, spleen & kidney	Illumina HiSeq 2000	114.5 M	SRX731259	[46]
17	Nile tilapia	*Streptococcus agalactiae*	Spleen	Illumina Genome Analyzer	91.4 M	NA	[47]
18	Giant grouper	*Vibrio alginolyticus*	Whole animal	Illumina HiSeq 2000	28.7 M *	NA	[48]
19	Mozambique tilapia	*Streptococcus agalactiae*	Spleen	Illumina HiSeq 2000	200 M *	SAMD00020886–SAMD00020893	[49]
		(with stimulated climate warming)					
20	Japanese sea bass	*Vibrio anguillarum*	Head kidney, liver & spleen	Illumina HiSeq 2000	334.3 M *	SRA296398	[50]
21	Grass carp	*Aeromonas hydrophila*	Spleen	Illumina HiSeq 2500	63 M *^,a^	SRR3045340; SRR3045341	[51]
22	Common carp	*Aeromonas hydrophila*	Spleen	Illumina HiSeq 2000	545 M	SRP072018	[52]
23	Nile tilapia	*Streptococcus agalactiae*	Spleen	Illumina HiSeq 2000	208 M	NA	[53]
24	Golden mahseer	*Aeromonas hydrophila*	Liver	Illumina NextSeq 500	Control: 25.7 M	SRS1117943; SRS1143332; SRS1152837	[54]
					Treatment: 27.5 M & 43.1 M		
25	Amur sturgeon	*Yersinia ruckeri*	Spleen	Illumina HiSeq 4000	Infected: 137.76 M	SRP110853	[55]
					Non infected: 139.49 M		
26	-	*Yersinia ruckeri*	-	Illumina NextSeq	250 M	NA	[56]
27	Grass carp	*Aeromonas hydrophila*	Intestine	Illumina HiSeq 2000	45.8 M *	SAMN04569528	[57]
28	Zebra fish	*Mycobacterium marinum*	Muscle	Illumina HiSeq 2500	NA	NA	[24]
29	Orange spotted grouper	*Vibrio harveyi*	Head kidney & spleen	Illumina HiSeq 4000	235.3 M *	NA	[58]
30	Yellow catfish	*Edwardsiella ictaluri*	Spleen	Illumina HiSeq 2000	Control: 50.9 M	SRX2746577; SRX2746578.	[32]
					Treatment: 51.4 M		
31	Japanese flounder	*Edwardsiella tarda*	Gill	Illumina HiSeq 4000	417.1 M	PRJNA359626	[33]
32	Hybrid tilapia	*Streptococcus agalactiae*	Liver, intestine & brain	Illumina HiSeq 2000	NA	NA	[59]
33	Channel catfish	*Flavobacterium columnare*	Gill	Illumina HiSeq 2000	23 M ^a^	SRP070957	[37]
34	Darkbarbel catfish	*Aeromonas hydrophila*	Spleen	Illumina HiSeq 3000	439.9 M *	PRJNA383309	[60]

* Clean reads; NA—Not available; M—Million reads; ^a^—Average read values.

**Table 2:** RNA-Seq analysis of fish in response to viral pathogens. This table shows a description of transcriptomes to understand viral diseases in various fish species.

Sl No	Fish	Virus	Sample	Sequencing Platform	Sequencing Depth/Library	Accession Number	Reference
1	Rainbow trout	Poly I:C (viral mimic)	Erythrocytes	ABI SOLiD3	80 M *^,a^	NA	[66]
2	Large yellow croaker	Poly I:C (viral mimic)	Spleen	Illumina Genome Analyzer II	56.3 M	SRP035897	[67]
3	Rainbow trout	Viral Hemorrhagic Septicemia Virus	Spleen & rayed fin	Illumina HiSeq 2000	3.119 B	NA	[68]
4	Grass carp	Grass carp reovirus	Gill, intestine, liver & spleen	Illumina HiSeq 2000	203 M	SRA099702	[69]
5	Atlantic salmon	Infectious salmon anaemia virus	Head kidney, liver & gills	Illumina MiSeq	196 M	SRX658605	[70]
6	Grass carp	Grass carp reovirus	Head kidney & spleen	Illumina MiSeq	107.9 M *	SRP049081	[71]
7	Miiuy croaker	Poly I:C (viral mimic)	Spleen	Illumina HiSeq 2000	Control: 50.3 M *	NA	[72]
					Treated: 48.2 M *		
8	Grass carp	Grass carp reovirus	Spleen	Illumina HiSeq 2000	Control: 2.72 M *	NA	[73]
					Infected: 2.75 M *		
9	Olive flounder	Heat inactivated viral hemorrhagic septicemia virus	Kidney	Illumina HiSeq 2500	Control: 23.7 M *	NA	[74]
					Day 1: 24.4 M *		
					Day 3: 23.8 M *		
					Day 7: 25.9 M *		
10	Atlantic salmon	Infectious salmon anaemia virus	Spleen	Illumina MiSeq	92.8 M *	NA	[75]
11	Schizothorax	Poly I:C (viral mimic)	Spleen	Illumina Hiseq 2500	Control: 53.8 M *	NA	[76]
					Treated: 55.1 M *		
12	Orange spotted grouper	Megalocytivirus and Ranavirus	Spleen	Illumina HiSeq	NA	NA	[77]
13	Grouper	Betanodavirus	Kidney cell line	Illumina HiSeq 2000	51 M	JR139431–JR139455	[78]
						JX644070	
14	Sockeye salmon	Piscine reovirus along with superinfection of infectious hematopoietic necrosis virus	Kidney	Illumina HiSeq 2000	2.3 B	SRP0740078	[79]
15	Atlantic salmon	Salmonid	TO-cells, a macrophage/dendritic like cell line	Illumina HiSeq 2000	Interferon treated: 23.1 M *	GSE64095	[80]
					SAV3 infection: 24.6 M *		
		alphavirus subtype-3			Control: 23.4 M *		
16	Gilthead seabream & European seabass	Nervous necrosis virus (Betanodavirus)	Leucocytes	Illumina HiSeq 2000	53 M ^a^	GSE101662	[81]
17	Koi carp	Cyprinid Herpesvirus 3	Spleen	Illumina HiSeq 2500	111.2 M	NA	[82]
18	Yellow catfish	Poly I:C (viral mimic)	Liver	Illumina HiSeq 2000	Control: 42.2 M *	NA	[83]
					Treated: 41.5 M *		
19	Zebrafish	Spring viremia of carp virus	Brain & spleen	Illumina HiSeq 4000	360.9 M *	NA	[84]
20	Olive flounder	Viral hemorrhagic septicemia virus	Kidney	Illumina HiSeq 2000	215.8 M	NA	[85]
21	Orange spotted grouper	Singapore grouper iridovirus	Spleen	Roche 454 Genome	Control: 0.428 M	SRA040065.1	[86]
				Sequencer FLX	Infected: 0.446 M		
22	Turbot	Megalocytivirus	Spleen	Illumina HiSeq 2000	S16-4d: 82.9 M	SAMN06186733 to SAMN06186736	[87]
					S16-8d: 86.5 M		
					S16-4d: 81.4 M		
					S16-4d: 79.1 M		

* Clean reads; NA—Not available; M—Million reads; B—Billion reads; ^a^—Average read values.

**Table 3:** RNA-Seq analysis of fish in response to parasites. This table shows a summary of investigations based on transcriptome analyses to understand parasitic diseases in various fish species.

Sl No	Fish	Parasite Species	Sample	Sequencing Platform	Sequencing Depth/Library	Accession Number	Reference
1	Large yellow croaker	*Enteromyxum scophthalmi*	Head kidney, spleen & pyloric caeca	Illumina HiSeq 2000	170 M	GSE63911	[107]
2	Three spined sticklebacks	*Diplostomum pseudospathaceum*	Head kidney & gills	Illumina HiScan SQ	486 M	PRJNA253091	[108]
3	_	*Caligus rogercresseyi*	Nauplius I, nauplius II, copepodid, chalimus, adult male & female	Illumina MiSeq	154.84 M	SRR1106551	[109]
4	Three-spined sticklebacks	*Diplostomum pseudospathaceum*	Head kidney & gills	Illumina HiScan SQ	990 M	PRJNA276419	[110]
5	Large yellow croaker	*Cryptocaryon irritans*	Liver	Illumina HiSeq 2000	Control: 51.9 M	NA	[111]
					Infected: 54.8 M		
6	Turbot	*Enteromyxum scophthalmi*	Spleen, head kidney & pyloric caeca	Illumina HiSeq 2000	170 M	PRJNA300347	[112]
7	Large yellow croaker	*Cryptocaryon irritans*	Gill, skin, spleen, head kidney & liver	Illumina MiSeq	49.5 M	NA	[113]
8	_	*Cryptocaryon irritans*	Tomont stage	Illumina MiSeq	80.8 M	NA	[114]
9	_	*Cryptocaryon irritans*	Trophonts, tomonts, and theronts stages	Illumina HiSeq 2500	Trophonts: 79.35 M	SUB1416064	[115]
					Tomont: 66.42 M	SUB1416075	
					Theronts:123.62 M	SUB1416142	
10	Orange spotted grouper	*Cryptocaryon irritans*	Skin	Illumina HiSeq 2500	506.6 M	GSE97397	[116]

* Clean reads; NA—Not available; M—Million reads.

**Table 4:** RNA-Seq analysis of various fish species. This table shows a list of studies based on transcriptome analyses to understand the immune system and its evolutionary significance in healthy fish.

Sl No	Fish	Sample	Sequencing Platform	Sequencing Depth/Library	Accession Number	Reference
1	Ayu	Macrophage	Illumina HiSeq 2000	27.96 M	SRA047923.1	[135]
					JP722270–JP772077	
2	Mud loach	Skin	Illumina Genome Analyzer II	111 M	SRA057415	[138]
3	European sea bass	Gills	Illumina HiSeq2000	68.6 M	NA	[133]
4	Fugu	Gills & swimbladder	Illumina HiSeq 2000	Gill: 55 M	SRA109280	[131]
				Swimbladder: 44.7 M	SRA109284	
5	Rainbow trout	Spleen	Illumina Genome Analyzer	93.5 M	NA	[126]
6	Japanese flounder	Spleen	Illumina MiSeq	14.6 M	SRR1515192	[2]
7	Naked carp	Gills & kidney	Illumina HiSeq 2000	Gill: 90.5 M	SRX673786	[127]
				Kidney: 90.4 M	SRX673788	
8	Tilapia	Brain, pituitary, gill, heart, liver, spleen, kidney, intestine, muscle, testis & ovary	Illumina Genome Analyzer	52.4 M	SRS676061	[132]
9	Topmouth culter	Brain, heart, gill, liver, intestines, muscle, gonads, kidney & pancreas	Illumina HiSeq 4000	44.9 M	SRX1502706	[129]
10	Grass carp	Spleen	Illumina HiSeq 4000	10.1 M	SRP078553	[130]
11	Tibetan Schizothoracinae	Gills & kidney	Illumina HiSeq 2000	Gill: 85.3 M	SRX673793	[128]
				Kidney: 88.7 M	SRX673788	
12	Zebrafish	Whole animal	Illumina HiSeq 2000	142.9 M	SRR4045953	[139]
13	Zebrafish	Kidney-derived blood cells	Illumina HiSeq 2500	1 M ^a^	E-MTAB-5530	[136]
					E-MTAB-4617	
					E-MTAB-3947	
14	Zebrafish	Spleen-derived lck:GFP cells	Illumina HiSeq 2000	2.1 M *^,a^	E-MTAB-4617	[137]

* Clean reads; NA—Not available; M—Million reads; ^a^—Average read values.

## Abbreviations

CAGE	Cap analysis gene expression
CyHV-3	Cyprinid herpesvirus-3
GALT	Gut-associated lymphoid tissue
GCRV	Grass carp reovirus
HSMI	Heart and skeletal muscle inflammation
IHNV	Infectious hematopoietic necrosis virus
ISAV	Infectious salmon anaemia virus
JAK	Janus kinase
KHV	Koi herpesvirus
LPS	Lipopolysaccharide
MHC	Major histocompatibility complex
NCBI	National center for biotechnology information
NGS	Next-generation sequencing
NLR	Nucleotide-binding oligomerization domain-like receptor
NNV	Nervous necrosis virus
PRV	Piscine orthoreovirus
RBL	Rhamnose-binding lectin
RLR	Retinoic acid inducible gene-I like receptor
RIN	RNA integrity number
RNA-Seq	RNA sequencing
SAGE	Series analysis of gene expression
SAV	Salmonid alphavirus
ScRNA-Seq	Single-cell RNA-Seq
SGIV	Singapore grouper iridovirus
SNP	Single nucleotide polymorphism
SSR	Single sequence repeat
SVCV	Spring viremia of carp virus
TLR	Toll-like receptor
VER	Viral encephalopathy and retinopathy
VHSV	Viral hemorrhagic septicemia virus
VNN	Viral nervous necrosis
